# Antioxidant properties and antimicrobial activity of selenium nanoparticles synthetized via Zambian medicinal herbs

**DOI:** 10.1371/journal.pone.0325460

**Published:** 2025-06-20

**Authors:** Pompido Chilala, Monika Jurickova, Zuzana Pokorna, Tereza Motlova, Pavel Horky, Sylvie Skalickova

**Affiliations:** 1 Department of Animal Nutrition and Forage Production, Faculty of AgriSciences, Mendel University in Brno, Brno, Czech Republic; 2 Department of Electron Microscopy, Institute of Scientific Instruments of the Czech Academy of Sciences, Brno, Czech Republic,; 3 Department of Microelectronics, Brno University of Technology, Technická 3058/10, 616 00, Brno, Czech Republic; Cairo University, Faculty of Science, EGYPT

## Abstract

Previous studies of green synthesized selenium nanoparticles (SeNPs) showed their unique properties such as antibacterial activity, biocompatibility, and antioxidant properties. This study aimed to use traditional Zambian medicinal herbs (*Azadirachta indica, Moringa oleifera Gliricidia sepium, Cissus quadrangularis, Aloe barbadensis, Kigelia Africana*, and *Bobgunnia madagascariensis*) to synthesize SeNPs and examine their potential to enhance the endogenous antioxidant system of model eukaryote. For SeNP characterization, dynamic light scattering, scanning electron microscopy, Fourier transform infrared spectroscopy,and absorbance spectra were used. Their minimal inhibitory concentration was investigated on *Staphylococcus aureus (S. aureus)* and *Escherichia coli (E. coli)* bacteria. The antioxidant potential of SeNPs was examined on *Saccharomyces cerevisiae (S. cerevisiae)*. Cell viability, total antioxidant capacity, and activity of superoxide dismutase, catalase, and glutathione peroxidase were evaluated. SeNPs did not show antimicrobial activity against *E. coli*, only mild activity against *S. aureus*. Experimental data suggested that SeNPs didn´t inhibit *Saccharomyces cerevisiae* growth while plant extracts and sodium selenite had an inhibitory effect. All tested plant extracts and SeNPs resulted in a significant decrease in superoxide dismutase activity compared to the control. Catalase activity significantly increased only in treatments with plant extracts or sodium selenite alone. Glutathione peroxidase activity remained the same for all studied SeNPs and plant extracts. These findings provide evidence of a complex influence of SeNPs or plant extracts on the cellular antioxidant system in *S. cerevisiae*. From the point of view of overall effectiveness, *Azadirachta indica, Moringa oleifera, Aloe barbadensis, and Cissus quadrangularis* SeNPs are promising, green-synthetized nanoparticles for combating oxidative stress in living organisms.

## 1. Background

The research community has been keenly interested in the role of selenium in animal and human health. Selenium has been proven to play a vital role in various critical metabolic pathways, such as immune and thyroid hormone metabolism functions. Selenium is also well known that it has one of the narrowest ranges between dietary deficiency dose (< 40 µg per day) and toxic levels (> 400 µg per day). The recommended intake by adults is 60 µg per day [1]. However, in several regions, such as Southern Africa [2], there is a selenium deficiency in the soil, which consequently leads to its low presence in the food supply, necessitating supplementation. Its deficiency is equally hazardous as its excess [3, 4].

With the development of nanotechnology, increasing attention has been directed towards the potential of selenium in nano-form to overcome the challenge posed by the narrow margin between its therapeutic efficacy and toxicity [5]. It is well known that the small dimensions of nanoparticles (1–100 nanometres) give them unique properties that have been used in many fields of human live [6]. In the last decade, the synthesis of nanoparticles (NPs) has been oriented towards ecological trends. An eco-friendly alternative to the chemical route is green synthesis which uses plant parts, enzymes, extracts, or microbes. Moreover, it is important to note that several studies have indicated that they pose better functionality and biocompatibility of NPs prepared by green synthesis compared to chemically synthesized NPs [7].

Selenium nanoparticles (SeNPs) exhibit several therapeutic effects in medical applications mainly attributed to their impressive biological properties as well as low toxicity, better reactivity, and efficiency in low dosages. Green synthesis of SeNPs using plant extracts has gained popularity in the scientific world due to the fact that it requires non-toxic solvents and moderate temperatures. The formation of SeNPs is a result of the reduction and stabilizing properties of components such as microbial enzymes and plant extracts [6, 8]. In this context, green synthesized SeNPs could act as antioxidant system enhancers in mammals. It has been shown that they are good free radical scavengers [9], improve wound healing [10] and can amend the oxidation-induced damage in the body and protect its organs against necrosis and dysfunction [11].

Herbal or medicinal plants have been utilized for the prevention and cure of diseases in many parts of the world for so many years now. Seven traditional Zambian medicinal herbs were used in this study for SeNPs green synthesis: *Moringa oleifera (M)* [12]*, Azadirachta indica (neem, N) [*13*], Gliricidia sepium (G)* [14]*, Aloe barbadensis (A)* [15]*, Cissus quadrangularis (veld grape, VG)* [16]*, Kigelia Africana (sausage tree, ST)* [17], and *Bobgunnia madagascariensis (snake been, SB)* [18]. In traditional Zambian medicine, *Moringa oleifera* is highly regarded for its nutritional and medicinal properties. Leaves, pods, and roots of this tree are used for treating various diseases including malnutrition, pods are also used in water purification and as food. Most Zambians believe that consuming moringa powder can increase energy levels and has anti-aging properties. Medicinal properties of moringa have been documented to originate from the presence of bioactive compounds such flavonoids and isothiocyanates which controls oxidative stress, inflammation and apoptosis [19], [20]. Neem leaves and oil are commonly used in traditional medicine and pest management in organic farming due to the presence of Azadirachtin [21].. The leaves are usually boiled to make a bitter drink which is used to treat malaria, skin diseases, and gastrointestinal problems. Neem encompasses bioactive compounds such as azadirachtin and nimbin which have antibacterial, antifungal, and anti-inflammatory effects [22]. These compounds disrupt microbial growth and modulate immune responses in an organism [23]. The sausage tree fruits are traditionally mashed and applied to the skin to treat eczema and psoriasis, while the bark is used to make infusions for rheumatism and arthritis. The sausage tree fruit is also believed to aid in penis enlargement and is highly sought by traditional healers for deworming blood increment, and chest problems. Sausage tree contains flavonoids and naphthoquinones with antimicrobial, anti-inflammatory and anticancer effects on animals and humans [24]. Veld grape is used widely in treating chickens and livestock against infections such as Newcastle disease in chickens and coughs in cattle. In humans, the plant gel is believed to heal wounds from fire and lightning. Veld grape contains flavonoids and triterpenes that were observed to promote bone healing, decrease inflammation and support antioxidant activity [25]. Gliricidia is known for its multipurpose use because of its properties of fixing nitrogen in the soil and is used in traditional medicine to treat wounds and skin infections for livestock. Gliricidia contains flavonoids and tannins that were documented to have antimicrobial, anti-inflammatory and insecticidal properties [26], [27]. Snake bean seeds and bark are used for their purported anti-inflammatory and anti-pest properties. It is believed by locals that the plant can help in treating snake bites and is sometimes used in protective rituals against snakes. Snake bean was reported to contain flavonoids, saponins and alkaloids which have antimicrobial, anti-inflammatory and insecticidal effects [28], [29]. Aloe Vera is highly regarded for its healing properties by the Zambian people. Aloe vera gel is used to treat burn wounds and skin irritations. Zambians view aloe vera as a plant for healing many ailments and it is sometimes used for rituals and cleansing by traditional healers. Aloe vera has been documented by many scholars to contain flavonoids, alkaloids, and saponins which have medicinal properties [30].

The objective of the current study was the synthesis of SeNPs via the green route from medicinal Zambian plant extracts and verify the hypothesis that they will improve the antioxidant system of the model eukaryotic organism *S. cerevisiae* more than the plant extracts alone or selenite. The study includes the synthesis of particles using extracts that have not been previously published: *Gliricidia sepium* (G-SeNPs)*, Cissus quadrangularis* (VG-SeNPs), *Aloe barbadensis* (A-SeNPs), *Kigelia Africana* (ST-SeNPs), and *Bobgunnia madagascariensis* (SB-SeNPs).

## 2. Materials and methods

### 2.1. Chemicals

Na_2_SeO_3_, ethanol, ABTS (2,2’-azino-bis(3-ethylbenzothiazoline-6-sulfonic acid), potassium persulphate, YPD (yeast extract peptone dextrose), MH media (Mueller-Hinton growth medium), and other chemicals unless noted otherwise were purchased from Sigma Aldrich (USA). *Moringa oleifera leaves* (M)*, Azadirachta indica leaves* (N)*, Gliricidia sepium leaves* (G)*, Aloe barbadensis leaves* (A)*, Cissus quadrangularis stems,* (VG)*, Kigelia Africana fruit* (ST), and *Bobgunnia madagascariensis seed pods* (SB) were collected in the natural forests of Kasisi Agricultural Training Centre in Lusaka, Zambia (15°15’08.6“S 28°29’13.0”E). Plant materials were dried and grinded in traditional mortar.. *S. cerevisiae* (ATCC 9763), *S. aureus* (ATC 25923), and *E. coli* (ATCC 25922) were obtained from the Czech Collection of Microorganisms, Faculty of Science, Masaryk University, Brno, Czech Republic. The pH value was measured using inoLab Level 3 (Wissenschaftlich-Technische Werkstatten GmbH; Weilheim, Germany). Deionized water underwent demineralization by reverse osmosis using the instruments Aqua Osmotic 02 (Aqua Osmotic, Tisnov, Czech Republic).

### 2.2. Plant extracts preparation

Firstly, we optimized solvent extraction to verify extraction method. Results are shown in supplementary material (S1 File). For all procedures: one gram of each plant powder was dissolved in 10 mL of each solvent: 100% dH_2_O, 30% aqueous ethanol (EtOH), and 50% aqueous EtOH in a 15 mL falcon tube. Plant samples were incubated at 22°C or 60 °C (Heating chamber, Binder, Germany) for 1 hr or 24 hrs with occasional shaking by hand. After incubation, samples were centrifuged (14 000 g, 10 min, MPW-350e, Poland), and supernatants were collected and filtered via a 0.2 µm nylon syringe filter (CHS Filterpure, Chromservis, Czechia). Supernatants were immediately analyzed or used for SeNP green synthesis. The solvent extraction with H_2_0, 22°C and 24h was chosen for all plant materials because of the highest or comparable yields of antioxidant capacity of the extract.

### 2.3. SeNPs green synthesis

SeNPs were prepared via sodium selenite reduction mediated by plant extracts. Protocol was adopted by research published previously [31] and the concentration of sodium selenite was chosen on the basis of non-toxic doses for *S. cerevisiae* [32]. One mL of plant extract was slowly added to a 9 mL solution of sodium selenite (10 mM) under continuous stirring on the magnetic stirrer. The mixture was covered by parafilm and let react at 22°C, 600 rmp, 24 hr. SeNPs were stored at 4 °C.

### 2.4. Total antioxidant capacity (TAC)

TAC method was adopted from George et al. [33]. Briefly, ABTS (38.4 mg) was dissolved in 10 mL potassium persulfate (2.5 mM) and the mixture was reacted in the dark at room temperature for 24h to prepare ABTS* + solution. This was diluted with 200 mM PBS buffer (pH 7.4) to obtain an absorbance of 0.7 AU. All wells of the microtitration plate were filled up with 50 µL of PBS. Fifty µL of each sample were pipetted to row A (The first column of each microtitration plate was left for standard – ascorbic acid 0.5 mg/mL) and ½ diluted into rows B-G by multichannel pipette. To each well, 100 µL of ABTS* + were pipetted and the microplate was covered with parafilm and incubated for 30 min at 22°C in the dark. Absorbance readings were performed at 745 nm. IC50 value was defined as the effective concentration at which the ABTS radical was scavenged by 50%. Results were expressed as ascorbic acid equivalent.

### 2.5. Spectral scanning and absorbance measurement

Samples for spectral scanning and absorbance measurements were placed in 96-well microtitration plates (Nunc™ MicroWell™ 96-Well Microplates, Thermofisher Scientific, USA). All measurements were performed at 22 °C on the Synergy HTX microplate reader (Synergy HTX, Biotech USA).

### 2.6. Particles size measurement

The average particle size and size distribution were determined by Delsa Max Core (Beckman Coulter, USA). The SeNPs water solution of 50 µL was put into a plastic cuvette and measured at a detector angle of 173°, the wavelength of 658 nm, the refractive index of 1.33, and a temperature of 22 °C. The acquisition of each measurement was set to 20 repetitions.

### 2.7. Scanning transmission electron microscope

The synthesized nanoparticles were inspected in a Scanning Transmission Electron Microscope (STEM). They were micropipetted onto a STEM grid covered with a thin holey carbon film (S-147–9 Finder Grid, Agar Scientific, USA), dried briefly using an infra-red lamp, and then observed in the Scanning Transmission mode in a Magellan 400L Scanning Electron Microscope (Thermo Fisher Scientific, Hillsboro, OR, USA). The inspection was performed at an electron beam energy of 30 keV, beam current of 25 pA, and using an insertable segmented STEM detector. This detector allows discerning particles that are strong scatterers due to their higher atomic number. As selenium has an atomic number of 34, it can be easily seen among other, relatively lighter, residues of organic matter.

The STEM observation verified that there are indeed present particles with distinctly geometric shapes and a higher atomic number. Their size varied quite widely, from aggregates of small particles of a few tens of nanometers in size, to rod-like particles roughly 520 nm long.

An attempt to determine the elemental composition of the nanoparticles by Energy-Dispersive X-ray Spectroscopy was also made but the sensitivity of the method was not sufficient to prove the presence of selenium.

### 2.8. Fourier transform infrared spectroscopy of SeNPs

FTIR-ATR spectra of green synthetized SeNPs were collected using an INVENIO-R FTIR Spectrometer equipped with a single-reflection diamond ATR accessory – A225/Q Platinum ATR module (Bruker Optic Inc., Billerica, MA, USA). One mL of the SeNPs solution was dried to a powder. A small amount of a solid sample or crystalline sodium selenite was put on the crystal and a fixed load was applied to the sample to ensure full contact with the diamond ATR. Plant extracts were pipetted on crystal, dispersed and dried using external fan according to technique described by Stepankova et al [34]. Before each measurement, background spectra were collected. Spectra were recorded at 25 °C from 5000 to 80 cm ⁻ ¹*** at a resolution of 2 cm ⁻ ¹. Each spectrum was acquired by merging 128 interferograms. Bruker OPUS software was used for IR spectra recording and processing, and JDXview v0.2 softwarewas used for further spectra evaluation (JDXview: https://homepage.univie.ac.at/norbert.haider/cheminf/jdxview.html).

### 2.9. Antimicrobial activity of SeNPs

Fifty µL of sterile Müller–Hinton (MH) broth (Oxoid, Hampshire, UK) was pipetted to the sterile 96-well microtitration plate (Nunc, Thermofisher Scientific, USA). The first row (A) was filled with 50 µL of tested samples in triplicates. Samples were diluted by half-dilution with a multichannel pipette. The last column was left as a negative control. As a positive control, gentamycin (300 µg/mL) was used. The bacterial culture (*S. Aureus and E. Coli*) was diluted to the fresh sterile (MH) broth to OD_600_ = 0.1 and then 100 × . To each well, 150 µL of the diluted bacterial suspension was pipetted. The inoculated microtitration plates were incubated for 24 h at 37 °C.

### 2.10. Yeast cultivation and SeNPs/extracts treatment

*S. cerevisiae* culture was cultivated in YPD agar (Oxoid, Hampshire, UK). Colonies were collected and resuspended in 10 mL of sterile YPD broth (Oxoid, Hampshire, UK). The turbidity of inoculated YPD broth was set to 0.1 AU (λ = 600 nm) and then diluted 100 × . Into sterile 15 mL falcon tubes were added 5 mL of inoculated YPD broth and 1 mL of SeNPs or extract (1:9 in water). As controls, 1 mL of sterile PBS or sodium selenite (10 mM), were simultaneously prepared. Samples were incubated at 37°C on a rotator (Roto-Therm, Benchmark Scientific, Germany), 40 rpm and 48 hrs. After incubation, falcon tubes were centrifuged, and pellets were collected and washed 3 times in PBS buffer.

A dose of 900 µL of lysis buffer (50 mM Tris with 0.9% NaCl, 1% SDS, pH 7.4) was added to 100 mg of pellet and the samples were homogenized (Polytron PT 1200 E, Kinematica, Switzerland) and centrifuged. Supernatants were collected and stored at −45 °C until analysis.

### 2.11. *S. cerevisiae* viability

To each well in a sterile microtitration plate (Nunc, Thermofisher Scientific, USA) pipetted 50 µL of sterile YPD broth. Fifty µL of SeNPs or extract (1:9) were added to wells in row A and then diluted ½ to rows B-G. Row H was set as a control. Inoculated YPD broth (100 µL) with *S. Cerevisiae* was added to each well. The yeast growth was monitored by Synergy HTX (Biotek, USA). The optical density reading was set at 600 nm and was monitored at time zero and then at each half-hour for 24 hr at 37 °C.

### 2.12. Antioxidant enzyme activity assays

Catalase; CAT (cat. No.: EIACATC, Invitrogen, USA), Superoxide dismutase; SOD (cat. No.: EIASODC, Invitrogen, USA) and Glutathione peroxidase; GPx (cat. No.: E-BC-K096-S, Elabscience, China) activity was analyzed using colorimetric kits according to manufacturer protocol.

### 2.13. Statistics

The experimental work was carried out in three independent experiments. Obtained data are presented as an average value. Results were analyzed using ANOVA and Scheffe’s Test. A significant result is considered at p < 0.05. Data were processed using MICROSOFT EXCEL® (USA).

## 3. Results

### 3.1. SeNPs characterisation

**Fig 1:**
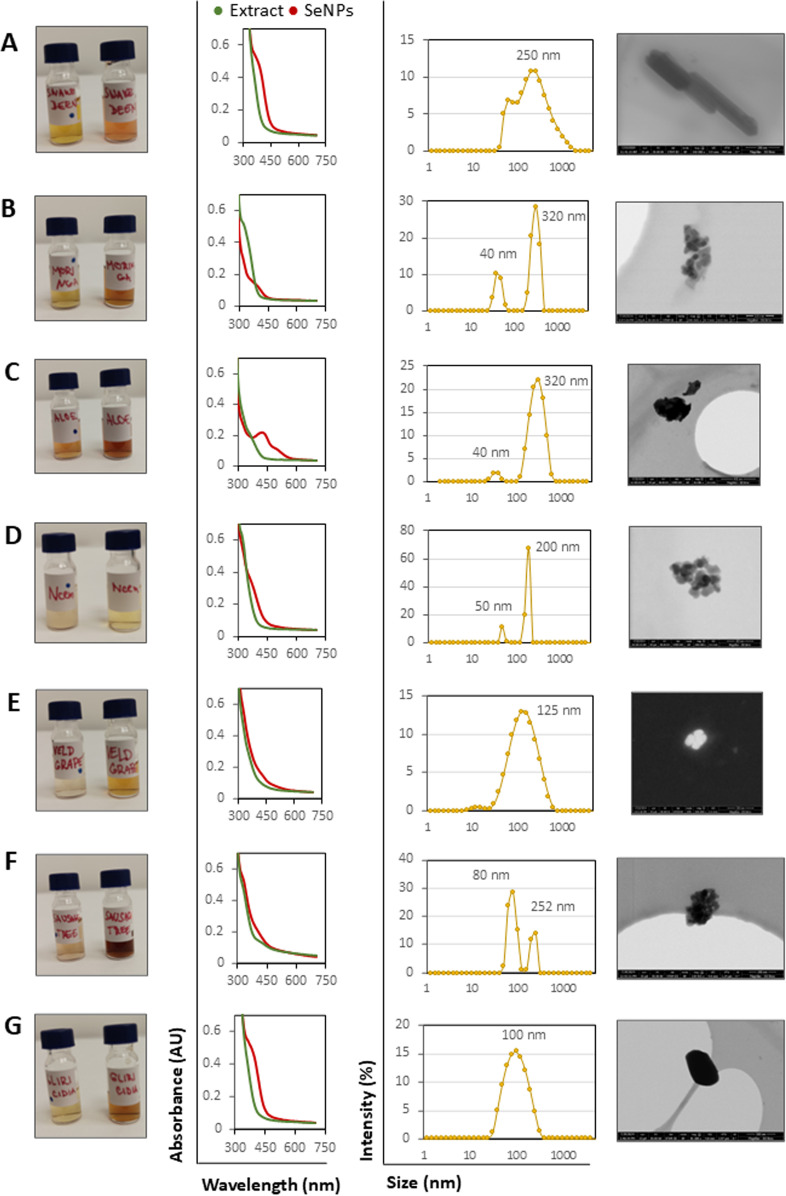
Characterization of green synthesized SeNPs. From the left: photo of plant extract and SeNPs (right), absorbance spectra, size distribution, and SEM photography. A) Snake bean, SB (*Bobgunnia madagascariensis*), B) Moringa, M (*Moringa oleifera*), C) Aloe, A (*Aloe barbadensis*), D) Neem, N (*Azadirachta indica*), E) Veld grape, VG (*Cissus quadrangularis*), F) Sausage tree, ST (*Kigelia Africana*), G) Gliricidia, G (*Gliricidia sepium*).

The formation of SeNPs was confirmed by recording absorption spectra, where the formation of nanoparticles causes a shift and change in the absorbance maximum. In several cases, this change was visible to the naked eye. The absorbance maximum and intensity vary based on the type of nanoparticles and fall within the range of 260–410 nm (Fig 1). The size of the nanoparticles is heterogeneous for each sample. Dynamic light scattering (DLS) showed that the nanoparticle size is heterogeneous, and smaller particles in the order of tens of nanometers are evident in SeNPs: SB, M, A, N, and ST. For all tested SeNPs, the size measured by DLS ranges from 50 to 320 nm (Fig 1).

TAC of SeNPs is shown in Fig 3A. The highest TAC was observed for SB-SeNPs (1500 µg/mL AA equivalent) and G-SeNPs (948 µg/mL AA equivalent) and the lowest TAC was measured in ST-SeNPs (193 µg/mL AA equivalent).

**Fig 2:**
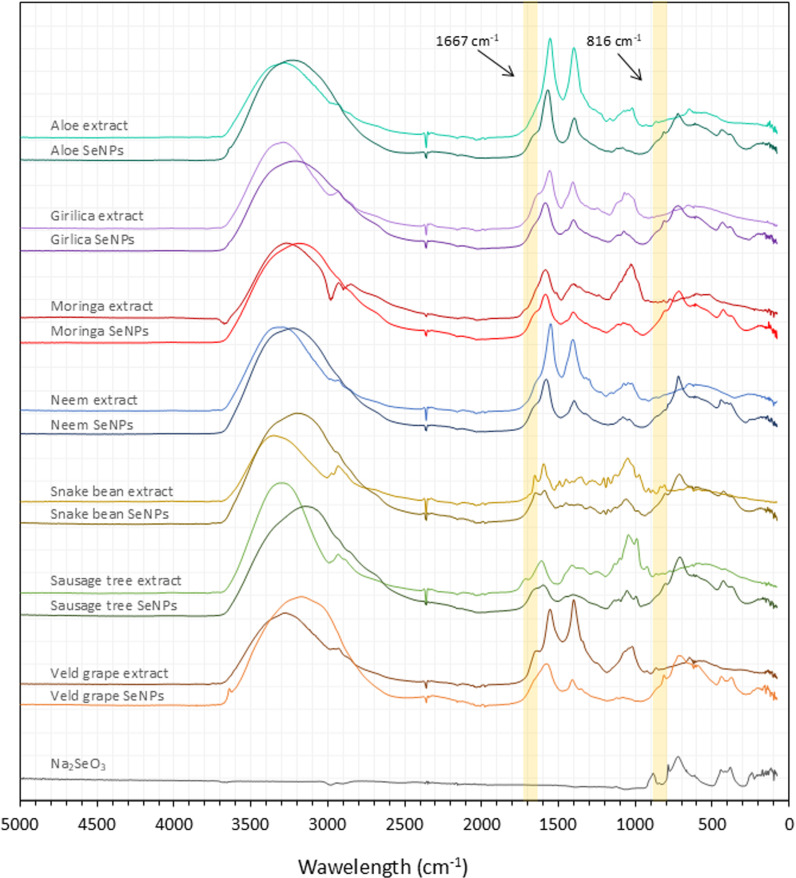
FTIR spectra of SeNPs, extracts and crystalline sodium selenite.

FTIR was employed to characterize the synthesized SeNPs and the corresponding plant extracts (Fig 2). The FTIR spectra of the SeNPs and plant extracts exhibited a broad absorption band around 3400 cm ⁻¹, which corresponds to the stretching vibrations of –OH groups typically associated with water molecules. This suggesting that the nanoparticles are likely present in a hydrated form.

The absorption band observed around 2937 cm ⁻¹ corresponds to C–H stretching vibrations and is present in all plant extracts, whereas it is absent in the SeNPs spectra. In the region above 1500 cm ⁻¹, characteristic absorption bands were observed in both the SeNPs and the plant extracts. These bands are primarily attributed to vibrations of aromatic and carbonyl functional groups commonly found in plant-derived organic compounds. Notably, spectral shifts were detected in this region in the SeNP samples, suggesting the formation of new chemical species or interactions between selenium and constituents of the plant extracts. All SeNPs samples displayed distinct absorption bands below 1000 cm ⁻ ¹, which are indicative of selenium-specific vibrational modes which is confirmed by absorption spectra of sodium selenite. A new absorption band appearing consistently around 816 cm ⁻ ¹ was identified in all SeNP samples.

### 3.2. Antimicrobial effect of SeNPs

Minimal inhibition concentrations of SeNPs and plant extracts are shown in Table 1. SeNPs as well as plant extracts and Na_2_SeO_3_ did not show an inhibitory effect on *E. coli*. VG-SeNPs and SB-SeNPs inhibited the growth of *S. aureus* at a concentration of 25% (*v/v*). The inhibitory effect on the growth *S. aureus* of M-SeNPs, A-SeNPs, N-SeNPs, ST-SeNPs, and G-SeNPs was at the concentration 50%. Na_2_SeO_3_ inhibits the growth of *S. aureus* at a 12% concentration (*v/v*).

**Table 1:** Minimal inhibition concentration (%) of plant extracts or green synthesized SeNPs. Each sample was tested in triplicates. ND = not detected.

		*E. coli*		*S. aureus*	
		Extract	SeNPs	Extract	SeNPs
**SB**	*Bobgunnia madagascariensis*	ND	ND	ND	25
**M**	*Moringa oleifera*	ND	ND	ND	50
**A**	*Aloe barbadensis*	ND	ND	ND	50
**N**	*Azadirachta indica*	ND	ND	ND	50
**VG**	*Cissus quadrangularis*	ND	ND	ND	25
**ST**	*Kigelia Africana*	ND	ND	ND	50
**G**	*Gliricidia sepium*	ND	ND	ND	50
	Na_2_SeO_3_	ND		12.5	

### 3.3. Viability of yeasts in the presence of SeNPs

**Fig 3:**
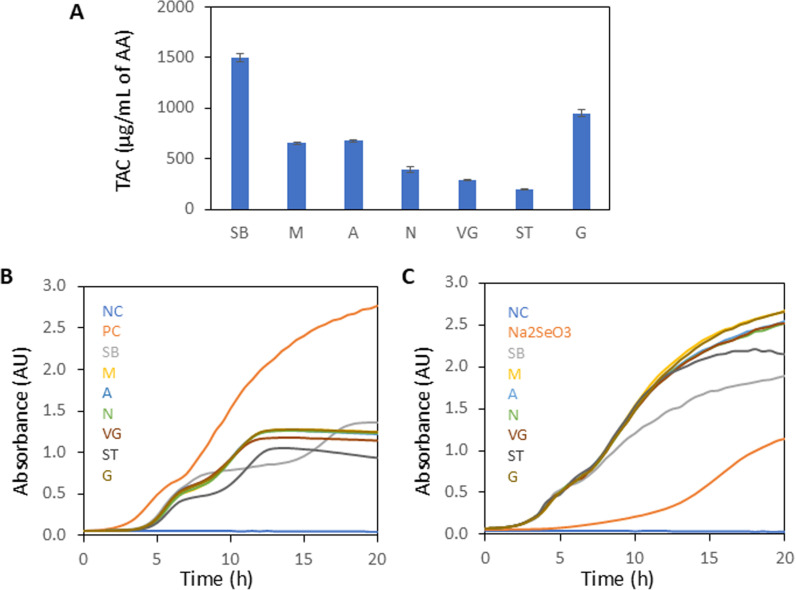
A) TAC of SeNPs. Viability of *S. cerevisiae*: B) Plant extracts, C) SeNPs. NC = negative control (pure medium), PC = positive control (*S. cerevisiae*), Na_2_SeO_3_ = *S. cerevisiae* with the addition of Na_2_SeO_3_ in the same concentration as SeNPs. **Legend:** Snake bean, SB (*Bobgunnia madagascariensis*), Moringa, M (*Moringa oleifera*), Aloe, A (*Aloe barbadensis*), Neem, N (*Azadirachta indica*), Veld grape, VG (*Cissus quadrangularis*), Sausage tree, ST (*Kigelia Africana*), Gliricidia, G (*Gliricidia sepium*).

The growth curves (Fig 3B, C) showed that the extracts accelerated the growth phase of yeast at the beginning of cultivation but slowed down the growth phase around the middle of the cultivation period. Additionally, viability decreased after 15 hours for all tested extracts except SB. For SB, the most rapid decline in viability was observed from 12 hours onwards. In the case of yeast cultivation with SeNPs, there is no evident rapid growth phase, and the growth curve mirrors the growth trend of negative control (NC). SeNPs treated *S. cerevisiae* remain viable even after 20 hours, except for ST-SeNPs, where growth is inhibited after 18 hours from the beginning of cultivation. For SB-SeNPs, yeast growth is slower compared to other SeNPs. It is also evident that *S. cerevisiae* with the addition of Na_2_SeO_3_ alone is inhibited in growth for up to 10 hours of cultivation.

### 3.4. Antioxidant effects of SeNPs on *S. cerevisiae*

**Fig 4:**
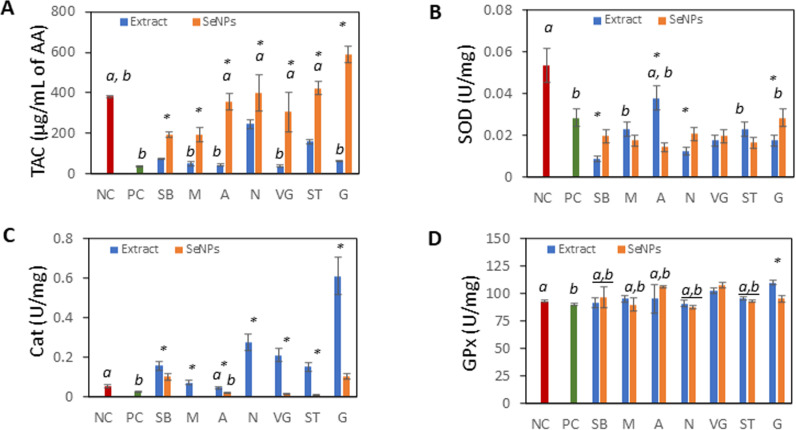
Antioxidant properties of *S. cerevisiae.* A) Total antioxidant capacity (TAC), B) Super-oxide dismutase (SOD), C) Catalase (CAT), D) Glutathion peroxidase (GPx). Samples: Snake bean, SB (Bobgunnia madagascariensis), Moringa, M (Moringa oleifera), Aloe, A (Aloe barbadensis), Neem, N (Azadirachta indica), Veld grape, VG (Cissus quadrangularis), Sausage tree, ST (Kigelia Africana), Gliricidia, G (Gliricidia sepium). Significance labeling: a – results are not significantly different from NC (p < 0.05); b – results are not significantly different from PC (p < 0.05); * – results of the extract and SeNPs are significantly different from each other (p < 0.05).

The impact of SeNPs on the endogenous enzymatic antioxidant defenses in *S. cerevisiae* was investigated. The overall antioxidant status (TAC) indicated that Na_2_SeO_3_ (positive control, PC) significantly reduces TAC compared to NC (*S. cerevisiae* without treatment) and SeNPs. Similarly, the addition of plant extracts alone significantly decreased TAC, with the greatest reductions observed for SB, M, A, VG, and G compared to SeNPs. In the case of SeNPs, TAC was significantly decreased by SB-SeNPs and M-SeNPs and increased by G-SeNPs compared to NC. All changes were statistically significant at p < 0.05. (Fig 4A).

Fig 4B shows that all treatments decreased SOD compared to NC (p < 0.05). Moreover, plant extracts SB, N, VG, G, and SeNPs except G-SeNPs caused a significant (p < 0.05) decrease in SOD activity compared to PC.

CAT activity was significantly reduced in PC, M-SeNPs, A-SeNPs, N-SeNPs, and VG-SeNPs compared to NC. Plant extracts SB, M, N, VG, ST, and G showed significantly increased CAT activity compared to NC. (Fig 3C). All changes were statistically significant at p < 0.05.

Glutathione peroxidase (GPx) activity was not affected by the extracts or SeNPs. However, significantly higher GPx activity in *S. cerevisiae* is observed with A and VG SeNPs, and VG and G plant extracts. All changes were statistically significant at p < 0.05

## 4. Discussion

A number of recent studies have shown that green-synthesized SeNPs are less toxic and more effective compared to inorganic selenium or its organic forms [35–38]. The objective of the study was to test the hypothesis that SeNPs synthesized from these plants enhance the antioxidant system of a eukaryotic cell model compared to the plant extracts or sodium selenite.

Compounds involved in green synthesis from plant extracts possess reducing capacity, which is crucial for NPs formation [39]. Not every extraction procedure was suitable for each plant powder, however, we chose extraction in water which showed good extraction yields for all tested plants, it is eco-friendly and cost-effective as well. Moreover, as Abubakar et al. [40] suggested, proper extraction solvent should be biocompatible and non-toxic which was crucial for further experiments. It has been previously shown that the ABTS method is suitable for evaluating the redox capacity of plant extract [41]. According to our results, the antioxidant capacity of plant extracts decreased in this order from the highest (30 µg/mL TAC) to the lowest (1 µg/mL TAC): SB – M – A – VG – N – ST – G. It has been shown that the antioxidant activity of plant extracts is influenced by interconnected factors such as phytochemical composition, part of plant, maturity, solubility of active compounds and assay method [42]. Based on further results, we did not demonstrate that the higher antioxidant capacity of plant extracts strongly affects the SeNPs size during their formation, as well as their antimicrobial activity and has any influence on the endogenous antioxidant system of *S. cerevisiae*.

The spectral characteristics, size, and shape of the SeNPs varied depending on the composition of the plant extracts used. Additionally, naturally present proteins and carbohydrates act as capping and stabilizing agents [43]. Also, herbal polyphenols act as selenium reducers and contribute to their antioxidant and antimicrobial properties [44]. When SeNPs formed, the samples exhibited a color change and absorption peak between 300–350 nm which is characteristic for SeNPs [45]. FTIR spectra showed a formation of new absorption band appearing consistently around 816 cm ⁻ ¹ was identified in all SeNP samples. Although relatively weak, the reproducibility of this band suggests that it may serve as a distinguishing feature for selenium nanoparticles. However in the existing literature, characteristic absorption bands attributed to elemental selenium (Se⁰) in green-synthesized nanoparticles have been reported at approximately 823 cm ⁻ ¹, 714 cm ⁻ ¹, 705 cm ⁻ ¹, 551 cm ⁻ ¹, and 447 cm ⁻ ¹ [46–49]. These bands, however, are typically associated with fingerprint region, where deformation vibrations of simple bonds are typically observed, making precise identification challenging in this context. The region range from 3730 to 400 cm ⁻ ¹ is often attributed to vibrational modes of functional groups involved in reduction, capping and stabilization of green synthetized nanoparticles [50]. Despite the expected relevance of this region for tracking reduction and stabilization processes, our FTIR spectra of SeNPs did not reveal clearly visible alterations or new features compared to SeNPs and corresponding extracts. Only disappearance of the absorption band around 1667 cm ⁻ ¹ in the plant extract is clearly observable, which may indicate that the corresponding compounds were either consumed or transformed during the reaction with selenite. The absence of detectable spectral change is likely due to the low intensity of SeNP-related signals, which are significantly overshadowed by the strong absorption bands from the plant extract matrix. Consequently, the overlapping and dominance of phytochemical signals hinder the clear resolution of bands specifically associated with elemental selenium or its interaction with bioactive molecules. Moreover, the SeNP spectra displayed absorption bands corresponding to both the plant extracts and selenite species. This observation indicates that a fraction of the initial selenite precursor remained unreacted. The incomplete reduction of selenite is likely attributable to insufficient extract concentration, which may have limited the reducing capacity during nanoparticle synthesis.

The extracts and their SeNPs did not show antimicrobial activity against G- E*. coli* but exhibited mild activity against G + *S. aureus*. This finding is contrary to previous studies that have demonstrated the strong antimicrobial potential of green-synthesized SeNPs. For instance, the study conducted by Khudier et al. [51] showed green synthesized SeNPs (10 mM Na_2_SeO_3_) from aloe vera extract with inhibition zones in cultivated *E. coli* and *S. aureus* around 10 mm diameter. Also, a study from Fardsadegh et al. [52] showed antimicrobial potential as well as the biocompatibility to L929 cell line (MTT test) of *Azadirachta indica* SeNPs (10 mM Na_2_SeO_3_). More evidence of the antimicrobial potential of green synthesized SeNPs could be found in review articles [53]. To support our findings, a chemical route of SeNPs (20 mM Na_2_SeO_3_) synthesis which enables controlled production of nanoparticles, has shown that antimicrobial activity relies on NPs chemistry, surface properties, size, and shape [54]. It has been shown that the mechanism of the antimicrobial effect of nanoparticles relies on the disruption of the bacterial cell wall and the induction of reactive oxygen species (ROS) which leads to bacterial death in which case the effectivity of these mechanisms depends on NPs chemical-physical nature [55]. These properties were not possessed by green synthesized SeNPs in this experiment.

*S. cerevisiae* as a eucaryotic cell model has been used in several studies to examine the effectivity of antioxidants [56–59]. Its metabolism and growth are not dependent on selenium intake, making it an ideal organism for investigating selenium toxicity and metabolism [60]. This study found that SeNPs did not inhibit *S. cerevisiae* viability while plant extracts and Na_2_SeO_3_ were mildly inhibitory on *S. cerevisiae* growth. These results align with studies confirming that selenium in the form of NPs is less toxic to eukaryotic cells compared to its inorganic form [38]. A previous study has found that the inhibitory dose of Na_2_SeO_3_ in *S. cerevisiae* is above 5 mM. Letavayová et al. [61] observed that inorganic selenium has toxic and mutagenic effects in stationary, and growth phases, whereas organic selenium in an equivalent dose is less harmful. The proposed mechanism of inorganic selenium toxicity is the production of ROS and dsDNA breakage. This could be explained by to less efficient uptake of selenate or only partial metabolic conversion to selenite and selenide [61]. Only SB-, and ST- SeNPs showed a slight inhibitory effect on *S. cerevisiae* growth. These two SeNPs differ from the other studied NPs in that SB-NPs and plant extract had the strongest antioxidant effect and ST is toxic at certain doses [17]. The reduced toxicity of SeNPs has been attributed to their controlled and gradual release of selenium, in contrast to the rapid ion release from sodium selenite [62]. Additionally, phytochemicals with reducing properties, present on the nanoparticle surface, exhibit antioxidant activities that may possess antioxidant activity, which can mitigate the pro-oxidative behavior and reactivity of SeNPs compared to sodium selenite in eukaryotic cells [63], [64]. Evidence of toxicity of studied plant extracts has been described in the literature [65–70], however synthetized SeNPs didn´t affect *S. cerevisiae* growth. Both extract and SeNP treatments of *S. cerevisiae* induced a shift in the balance of endogenous ROS and the cell’s antioxidant defenses. While the TAC of *S. cerevisiae* significantly decreased with plant extracts and sodium selenite treatment (PC), the TAC with SeNPs, except for SB and M, remained comparable to that of the untreated yeast cells (negative control, NC). A substantial decrease in TAC indicates enhanced endogenous ROS production. Moreover, inorganic Na_2_SeO_3_ and plant extracts could induce oxidative stress which depletes antioxidant defense [71]. However, it was found that some medicinal plants have protective effects against oxidative stress [72], their toxic effects or their bioactive molecules can behave as prooxidants [73].

SOD is the primary enzyme that mitigates ROS by converting superoxide radicals into hydrogen peroxide. All treatments in our experiment resulted in a decrease in SOD activity compared to the NC. Current findings indicate that reduced SOD activity indicates ongoing oxidative stress or inhibition of ROS by exogenous antioxidants [74]. The imbalance between production and accumulation of ROS in the cell promotes the activity of CAT. Results of this study confirmed higher CAT activity in and PC, but not in NC and SeNPs, except for SB-SeNPs and G-SeNPs. Lower CAT activity could be explained by lower presence of H_2_O_2_ as a product of SOD activity and metabolic processes. The hypothesis confirms Martins et al [75]. The CAT activity is induced by the H_2_O_2_ challenge of cells in a nutrient-rich YPD medium and protects against H_2_O_2_ stress. Glutathione peroxidase (GPx) activity remained unchanged with both; extracts and SeNPs compared to the NC, except for an increase in activity with the VG extract, G extract, and A-SeNPs. The unchanged GPx activity, alongside increased catalase activity, suggests a targeted response where catalase is upregulated to manage the specific increase in hydrogen peroxide, while GPx remains unaffected. The explanation could rely on the preferable elimination of H_2_O_2_ by CAT [55].

The selective modulation of enzyme activities indicates a complex interaction between SeNPs, plant extracts, and the cellular oxidative stress mechanisms in *S. cerevisiae*. From the point of view of overall effectiveness, N, M, A, and VG SeNPs seem to be promising nanoparticles for further studies because they can support the endogenous antioxidant system in eucaryotic cells and don´t disrupt their homeostasis. It should be emphasized that inorganic selenium and plant extracts of medicinal herbs can burden an antioxidant system of a cell and retard growth as we demonstrated in a model eukaryote *S. cerevisiae*. Further research should be aimed at elucidating the mechanisms that influence the green synthesized selenium nanoparticles biocompatibility as well as optimization of synthesis to highlight their antioxidant potential.

## 5. Conclusion

In our study, we confirmed that the formation of SeNPs and their size does not depend on the total antioxidant potential of the plant extracts. At the tested levels, SeNPs do not have an inhibitory effect on the growth of *S. cerevisiae* culture, while plant extracts and Na_2_SeO_3_ alone in an equimolar concentration do inhibit its growth. SeNPs N and then M, A, and VG support the antioxidant homeostasis of *S. cerevisiae* and have the potential to play a significant role in oxidative stress protection or adaptation. Future research could focus on optimizing the synthesis of green-synthesized SeNPs, as well as elucidating their surface properties and composition. Further investigations should also address the relationship between these characteristics and the observed antioxidant activity, the limited antimicrobial effect, and the potential impacts on mammalian cells.

## Supporting information

S1 File Plant extraction optimization. (DOCX)
