# Supplementary material for: Antioxidant properties and antimicrobial activity of selenium nanoparticles synthetized via Zambian medicinal herbs
Source: PLoS One. 2025 Jun 20;20(6):e0325460. doi: 10.1371/journal.pone.0325460 (PMC12180647; doi:10.1371/journal.pone.0325460)
Supplement: S1 File — (DOCX) [file pone.0325460.s001.docx]

**S1: Plant extraction optimization**

Due to the different chemical compositions of plants, the efficiency of extraction varied based on the selected procedure. For the study, extraction methods were evaluated based on the antioxidant capacity of the extract. Overall, the highest TAC yields were observed for SB, M, A, and VG while the lowest were for N, ST, and G, respectively. Results are graphically summarized in Fig. S1.


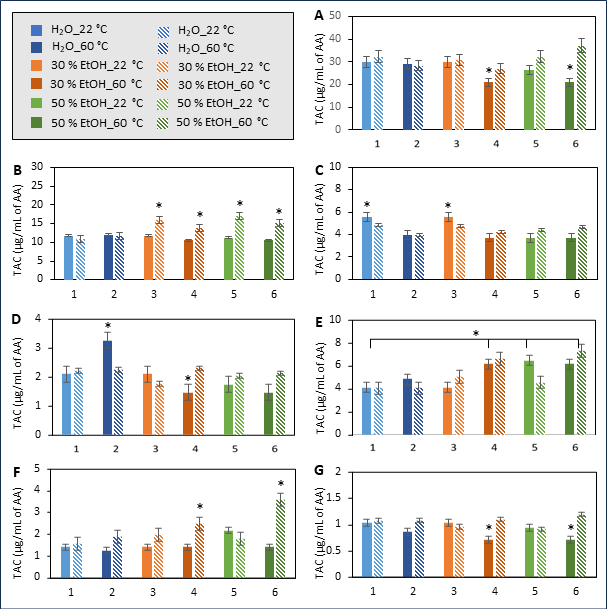


**Figure S1**: Influence of extraction method on total antioxidant capacity (TAC). TAC is expressed as an ascorbic acid equivalent. **A**) Snake bean, SB (*Bobgunnia madagascariensis*), **B**) Moringa, M (*Moringa oleifera*), ***C****)* Aloe, A (*Aloe barbadensis*)*,* ***D)*** Neem, N (*Azadirachta indica*)*,* ***E)*** Veld grape, VG (*Cissus quadrangularis*), ***F)*** Sausage tree, ST (*Kigelia Africana*) ***G)*** Gliricidia, G (*Gliricidia sepium*)*.*
